# A Cyclic {Ni^II^_8_Dy^III^_4_} Cluster Where the Dy^III^ Moments Are Organized by the Transition Metal Spins

**DOI:** 10.1002/chem.71499

**Published:** 2026-08-07

**Authors:** Imon J. Dutta, Kartik Chandra Mondal, Jonas Braun, Wolfgang Wernsdorfer, Christopher E. Anson, Yanhua Lan, Kuduva R. Vignesh, Annie K. Powell

**Affiliations:** ^1^ Department of Chemical Sciences Indian Institute of Science Education and Research (IISER) Mohali Mohali Punjab India; ^2^ Department of Chemistry Indian Institute of Technology Madras Chennai India; ^3^ Institute of Inorganic Chemistry Karlsruhe Institute of Technology (KIT) Karlsruhe Germany; ^4^ Institute of Nanotechnology Karlsruhe Institute of Technology (KIT) Karlsruhe Germany; ^5^ Institute for Quantum Materials and Technologies Karlsruhe Institute of Technology (KIT) Karlsruhe Germany; ^6^ Physikalisches Institut Karlsruhe Institute of Technology (KIT) Karlsruhe Germany

**Keywords:** 3d–4f complex, *ab initio* calculations, coordination chemistry, MicroSQUID, single molecule magnets

## Abstract

A series of [Ni_8_Ln_4_(CO_3_)_4_(L)_8_(NO_3_)_2_(H_2_O)_8_ (MeOH)_4_](NO_3_)_2_ (Ln = Dy (**1**), Gd (**2**), Y (**3**)) cyclic coordination clusters containing four carbonate bridges were isolated and studied using SQUID and microSQUID experiments and quantum mechanical calculations. The dodecanuclear Ni_8_Dy_4_ complex exhibits slow relaxation of the magnetization confirmed by the presence of temperature‐ and field‐dependent magnetic hysteresis loops. The competing interactions and the anisotropic nature within the cluster were analyzed using DFT and ab initio calculations and revealed that the spin structure of the Dy^III^ ions is steered by the interactions mediated through the Ni^II^ ions.

## Graphical abstract

The synthesis, structural characterisation, and magnetic properties of a cyclic Ni_8_Dy_4_ coordination cluster are reported. *Ab initio* CASSCF and DFT calculations help rationalize the shape of the microSQUID hysteresis and the competing magnetic interactions within the compound.

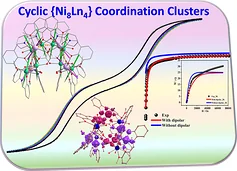

## 1 Introduction

Organization of spins in homometallic Dy^III^ clusters can lead to various magnetic effects such as single‐molecule magnetism (single‐molecule magnets—SMMs) or toroidicity (single molecule toroics—SMTs). The spin structures in such compounds are generally driven by dipolar couplings between Dy^III^ centers [1, 2, 3, 4]. However, in heterometallic 3d–4f systems, the situation can be more complicated, particularly in cases where the Dy^III^ ions are too far apart for direct dipolar Dy–Dy coupling to be significant. Here, the coupling of the spins of the intervening 3d ions with the Dy^III^ ions can organise the Dy^III^ moments to give exotic spin structures. Particularly, when these anisotropic Dy^III^ ions are coupled with anisotropic 3d transition metal ions such as Fe^II^, Co^II^, Ni^II^ and Mn^III^, they exhibit substantial zero‐field splitting (*D*); this results in strongly coupled spin systems. This has, for example, been shown in an Fe_8_Dy_12_ SMM [5] as well as an Fe_18_Dy_6_ SMT [6] and also in Fe_8_Dy_8_ [7], Mn_8_Dy_8_ [8], and Cr_8_Dy_8_ [9] wheels. Ni^II^ has been less frequently used as the 3d heterometal in 3d–4f clusters. Though the majority of Ni^II^ complexes have *D* values falling within the −200 to +200 cm^−1^ range [10, 11], which is usually observed for mononuclear Fe^II^ and Co^II^ complexes, a few Ni^II^ complexes possess a *D* value over −400 cm^−1^ [12, 13, 14]. For example, a trigonal bipyramidal Ni^II^ complex shows a *D* value of ≈ −535 cm^−1^ due to the lack of axial symmetry breaking [15, 16]. Therefore, the selection of Ni^II^ as the 3d metal is motivated by its substantial magnetic anisotropy, which arises from second‐order perturbative mixing of excited states and leads to pronounced *D* [10, 15, 17, 18]. Ni^II^–Ln^III^ complexes are attractive because they combine the large magnetic anisotropy of Ln^III^ ions with the ability of Ni^II^ to mediate appreciable exchange coupling [17]. Moreover, promising observations such as the identification of ferromagnetic coupling between Ni^II^–Ln^III^ ions suggest a beneficial synergy within 3d–4f complexes, significantly reduces the QTM and offering great potential SMMs with long relaxation times (>100 s) [19, 20, 21, 22, 23, 24]. Examples where it has been used include Ni_2_Dy_2_ [25], Ni_3_Dy_3_ [26], Ni_4_Dy_2_ [27], Ni_10_Ln_5_ [28], and Ni_22_Gd_44_ [29] clusters.

Here we report a cyclic dodecanuclear heterometallic {Ni_8_Dy_4_} complex [Ni_8_Dy_4_(CO_3_)_4_(L)_8_(NO_3_)_2_(H_2_O)_8_(MeOH)_4_](NO_3_)_2_·5MeOH·4H_2_O (**1**) obtained from a methanolic solution of Dy^III^:Ni^II^:H_2_L:Et_3_N in a yield of 30%–40% (H_2_L = (E)‐2‐(2‐hydroxy‐3‐methoxybenzylideneamino)phenol). The Gd^III^ (**2**) and Y^III^ (**3**) analogs were also synthesized using the same synthetic procedure. The magnetic SQUID and microSQUID data of these three isostructural compounds are described and rationalized by *ab initio* CASSCF and DFT calculations. Furthermore, the role of dipolar coupling in this large heterometallic {Ni^II^_8_Dy^III^_4_} cluster has been verified with DFT/PHI calculations. The important role of dipolar coupling in lanthanide‐containing clusters [30, 31] and in the lanthanide radical system has been explored [32]. In lanthanide radical complexes, the exchange coupling between a lanthanide ion and an organic radical is frequently stronger than the exchange between two lanthanide centers. These lanthanide‐radical exchange interactions are also comparable to typical 3d‐lanthanide exchange interactions. Therefore, we thoroughly calculate dipolar contributions for all relevant metal‐metal pairs (Ni–Dy and Ni–Ni) and compare these terms with the exchange parameters. This study highlights the crucial role of dipolar interactions in accurately reproducing the experimental dc magnetic data and in determining the SMM properties.

## 2 Results and Discussion

### 2.1 Crystallography

Compound **1** crystallizes in the tetragonal space group P $\bar{4}$2_1_ *c* with *Z* = 2, and the [Ni_8_Dy_4_(*μ*_5_‐CO_3_)_4_(L)_8_(MeOH)_4_(NO_3_)(OH_2_)_10_]^3+^ cluster molecule occupies a site with crystallographic $\bar{4}$ site symmetry (Tables S1 and S2). The asymmetric unit of the cluster and a view of the complete molecule are shown in Figure 1, with two views of the cluster core in Figure 2.

**FIGURE 1:**
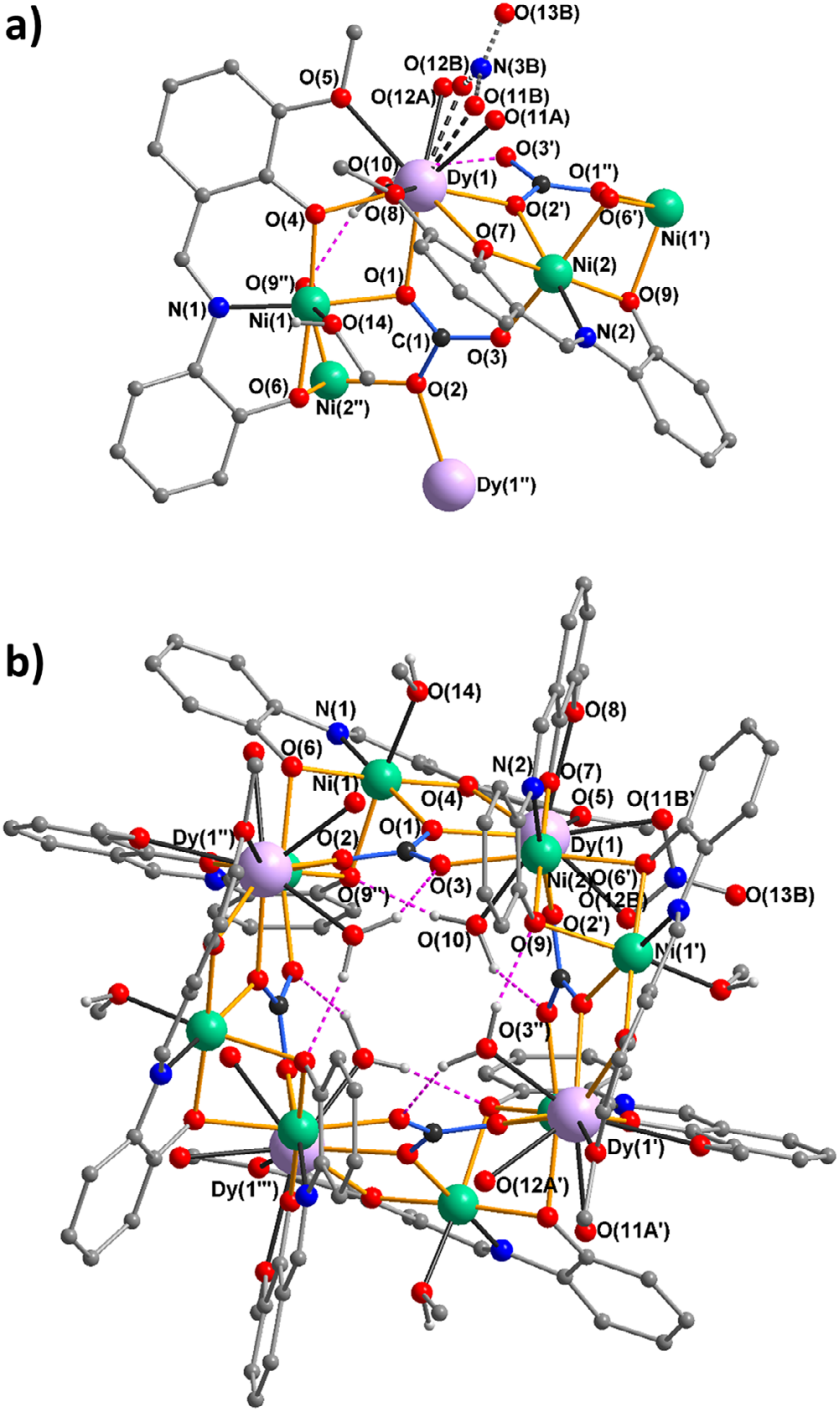
(a) Asymmetric unit of the [Ni_8_Dy_4_(*μ*_5_‐CO_3_)_4_(L)_8_(MeOH)_4_(NO_3_)(OH_2_)_10_]^3+^ cluster in **1**, showing the connections to symmetry‐equivalent metal atoms. For the disordered ligands on Dy1, the minor (25%) nitrate is shown with dashed bonds; the corresponding water ligands (occupancy 75%), O(11A), and O(12A), are shown with solid Dy─O bonds. (b) View of the complete cluster molecule viewed down the crystal *c*‐axis, with the disordered nitrate ligand shown in one of its four possible positions (chelating Dy(1) at the top right of the molecule as shown). Dy violet, Ni green, O red, N blue, C grey, and organic H‐atoms omitted for clarity. Hydrogen bonds shown as purple dashed lines; carbonate C─O bonds emphasized in light blue. Primed atoms at {*y*, −*x*, 1−*z*}, double‐primed atoms at {−*y*, *x*, 1−*z*}, and triple‐primed atoms at {−*x*, −*y*, *z*}.

**FIGURE 2:**
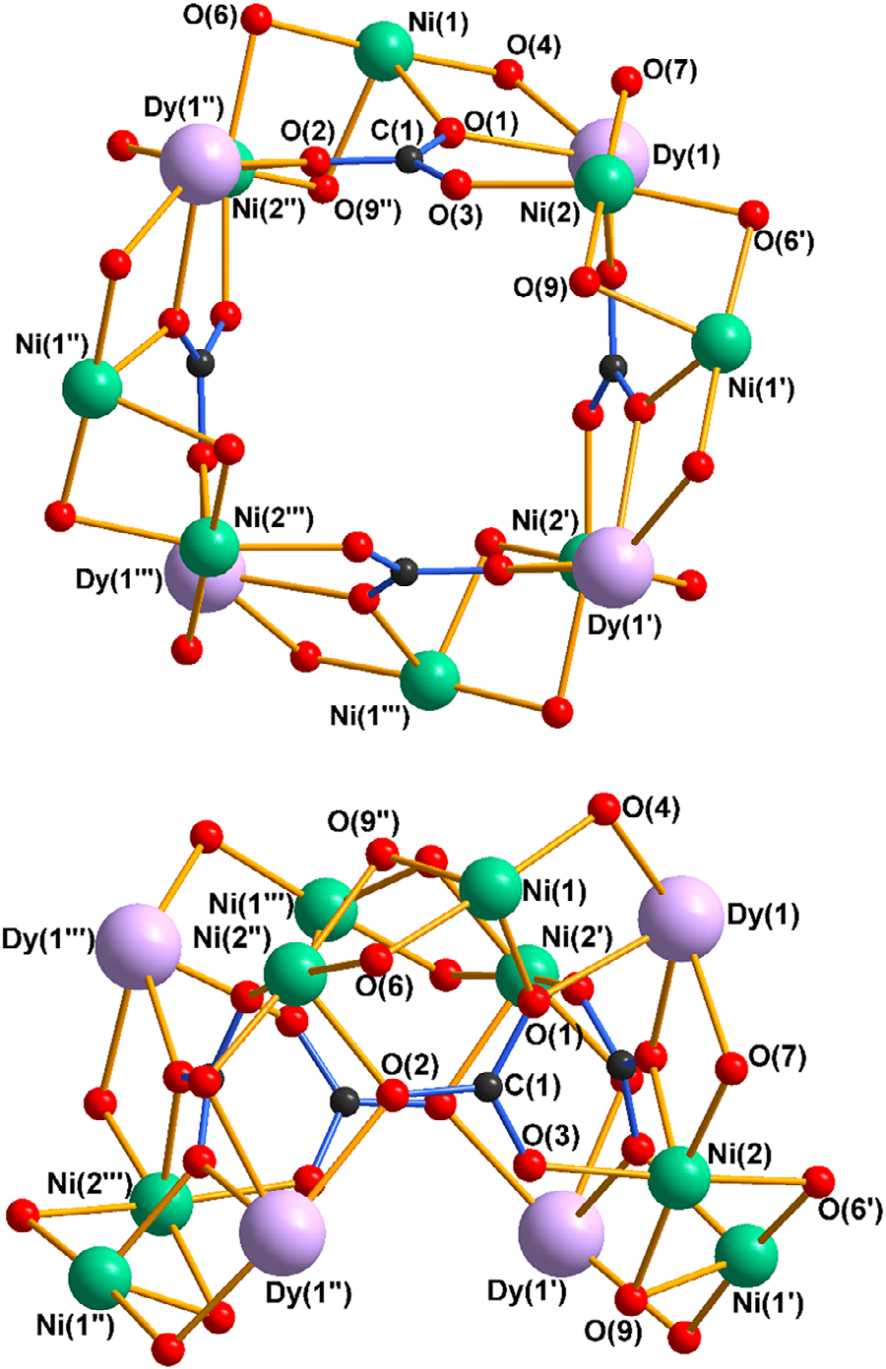
Two views of the Ni_8_Dy_4_ core in **1**, showing the carbonate and phenoxide bridges between the metal atoms. Atom colors and symmetry codes as for Figure 1.

The core of the cluster can be considered to be made up of four nonlinear Dy‐Ni‐Ni subunits, arranged alternately above and below the Ni_8_Dy_4_ mean plane, resulting in a highly nonplanar cyclic system. The organic ligands (L)^2−^ are the doubly deprotonated forms of the Schiff base formed between *o*‐vanillin and 2‐aminophenol, and as expected, the Ni^II^ ions occupy Pocket I of the ligand, made up of the two phenoxido oxygens and the imino nitrogen, whereas the methoxido group and phenoxido oxygen of the vanillin (Pocket II) chelate a Dy^III^. Each pair of adjacent Ni^II^ in the cycle is then bridged by two oxygens from aminophenol sub‐units, giving the bridging coordination mode shown in Scheme 1. Additional linkages between the metal centers in the ring are provided by the four carbonate bridges, each of which coordinates to three Ni^II^ and two Dy^III^ (Scheme 1, Figure 2). Further intramolecular stabilization is provided by the four water ligands O(10), which each make a hydrogen bond to a phenoxido oxygen and an oxygen of a carbonate bridge.

**SCHEME 1:**
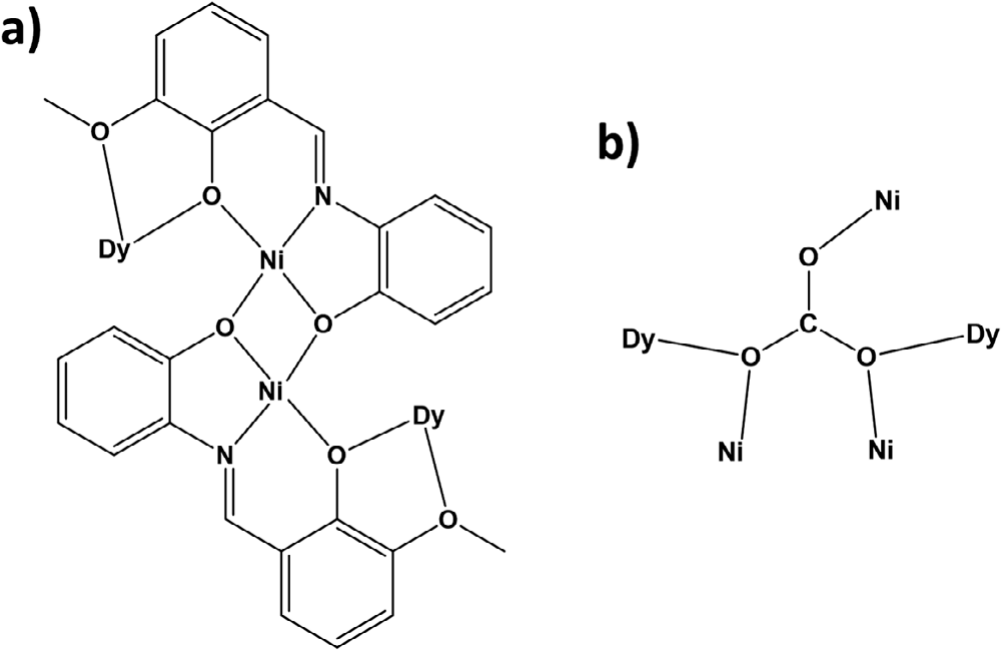
**(a)** Coordination mode of two of the ligands (L)^2−^ on either side of a Ni_2_ dinuclear unit within the Ni_8_Dy_4_ cluster in **1**. (b) Bridging mode of the (*μ*_5_−CO_3_)^2−^.

Peripheral coordination for the four Dy^III^ is provided by either a chelating nitrate (on one Dy^III^) or two further water ligands (the other three Dy^III^). There is no ordering of the molecules in the crystal in terms of the orientation of the single nitrate ligand, resulting in the apparent $\bar{4}$ site symmetry of the cluster. This fourfold symmetry results from the averaging of the coordination environments of the four Dy centers over the crystal as a whole. This means that in the asymmetric unit there is a 25:75 disordered superposition (Figure 1a). The coordination sphere of Ni(1), on the other hand, is completed by an ordered methanol ligand.

Overall, the cluster is thus a trication, and this charge is balanced by three lattice nitrates. Two of these are provided by the nitrate centered on the atom N(41), which occupies a site of twofold symmetry (thus two nitrates per cluster), whereas the third nitrate is disordered over the other $\bar{4}$ site in the structure. The primary intermolecular linkage involves the methanol ligands on four of the Ni^II^ centers, which form hydrogen bonds to the oxygen O(41) of the nitrate on the twofold site. This hydrogen bonding thus links each cluster to four other symmetry‐related clusters at {−*x*, 1−*y*, *z*}, {1−*y*, *x*, 1−*z*}, {*x*, −1+*y*, *z*}, and {−1+*y*, −*x*, 1−*z*}, resulting in undulating “chessboard” sheets parallel to [001]. The space group P $\bar{4}$2_1_ *c* is non‐centrosymmetric, and the essentially zero Flack *χ* parameter (−0.037(4)) shows the absence of any twinning of the structure by inversion.

Such incorporation of CO_3_^2−^ derived from atmospheric CO_2_ has also been observed in several Ni^II^ [33], Dy_6_ [34], Ln_10_ [35], NaNi_5_La_4_, and Ni_30_La_20_ [36] complexes. It is worth noting that when the same reaction was performed under inert atmosphere, it resulted in completely different products [25].

In terms of magnetic coupling, considering the molecular structure of complex **1**, the following can be expected. Ni─Ni interactions within each dimeric Ni^II^_2_ unit are expected to be ferromagnetic since the Ni‐phenoxide‐Ni angle of 88.13°(1) is smaller than the suggested critical angle of 96° [37]. The Ni─O─Dy bridging angle is equal to 103.46° in complex **1**. Furthermore, Ni_2_Dy_2_ sub‐units of **1** are also further magnetically coupled to alike neighboring units by additional *syn‐anti/anti‐anti* bridging modes (Figure S1c) of CO_3_^2−^ which are reported to mediate weak antiferromagnetic interactions [33, 36]. Each cationic molecular unit of **1** is involved in intermolecular H‐bonding, which could additionally mediate weak antiferromagnetic interactions (Figure S2).

### 2.2 Experimental Magnetic SQUID Data

The *χT* values at room temperature are 65.93 cm^3^ K mol^−1^ for **1**, 40.65 cm^3^ K mol^−1^ for **2**, and 8.36 cm^3^ K mol^−1^ for **3**, and are in good agreement with the expected values for compounds made of eight Ni^II^ ions (*S* = 1, *g* = 2.1, *C* = 1.10 cm^3^ K mol^−1^) and four Dy^III^ ions (*S* = 5/2, *L* = 5, ^6^H_15/2_, *g* = 4/3, *C* = 14.17 cm^3^ K mol^−1^), four Gd^III^ ions (*S* = 7/2, *L* = 0, ^8^S_7/2_, *g* = 2, *C* = 7.88 cm^3^ K mol^−1^), of 65.48, 40.3, and 8.8 cm^3^ K mol^−1^, respectively. The *χT* product for **1** remains essentially constant from room temperature to 100 K, below which it decreases slowly, followed by a sharp drop to 17.51 cm^3^ K mol^−1^ at 1.8 K (Figure 3, top). The *χT* versus *T* plots of **2** and **3** follow a similar profile with a gradual increase down to 100 K, below which the *χT* product of **2** increases more rapidly, reaching a maximum of 44.6 cm^3^ K mol^−1^ at 11 K, then sharply decreasing to 31.7 cm^3^ K mol^−1^ at 1.8 K. This is indicative of the presence of a dominant ferromagnetic exchange interaction (Figure 3, bottom). For **3,** the gradual increase continues down to 55 K and then sharply decreases to 0.78 cm^3^ K mol^−1^ at 1.8 K (Figure 3, bottom).

**FIGURE 3:**
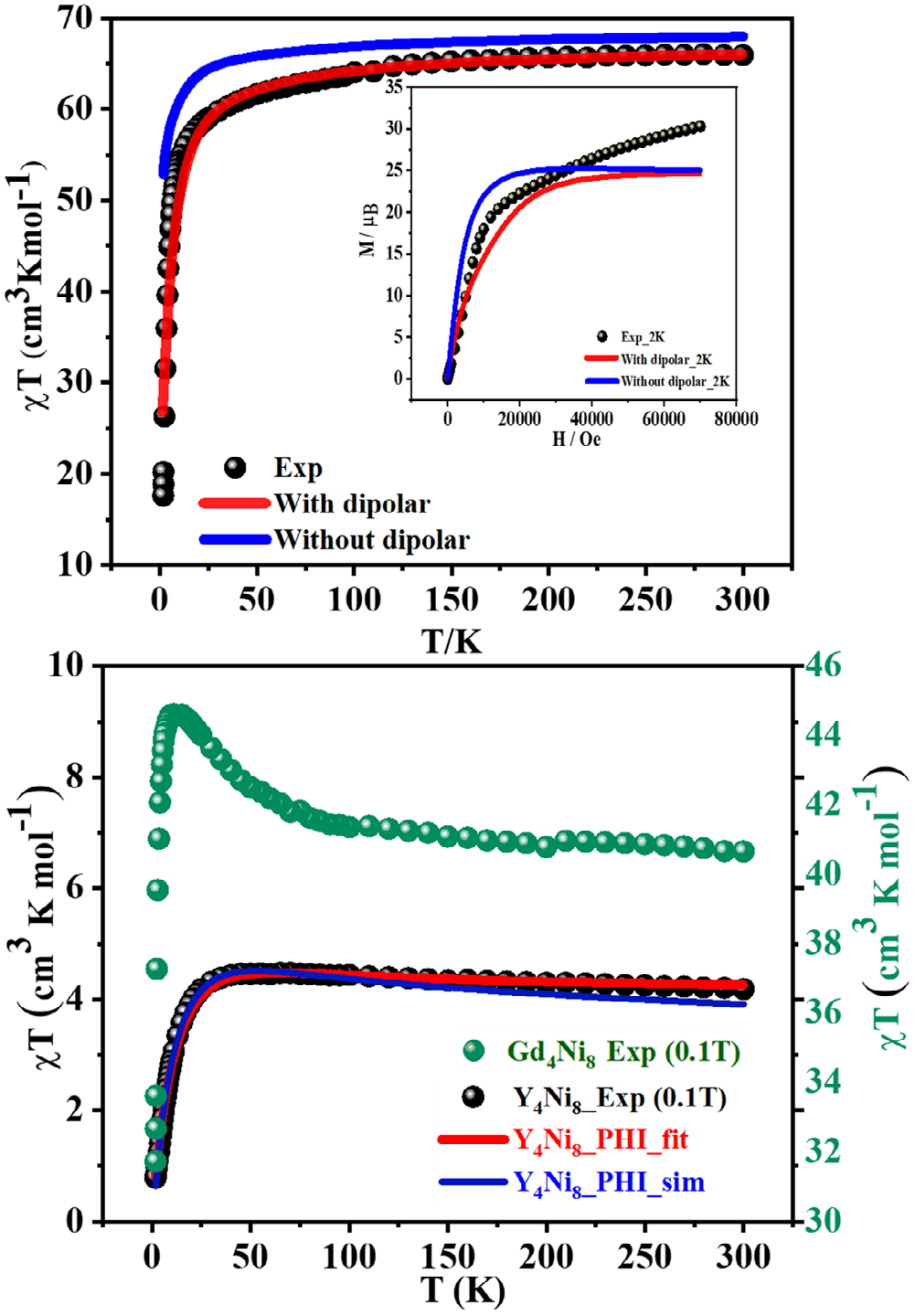
The *χT* vs. *T* and *M* vs. *H* at 2 K (inset) plots of complex **1** (top). The *χT* vs. *T* plot of complexes **2** and **3** (bottom). The curves for **3** obtained from the PHI fitting and simulated using the parameters from the DFT calculations are shown as red and blue lines, respectively.

The magnetization curves of **1** increase rapidly on increasing the dc magnetic field (*H*) below 1 T, above which *M* linearly increases up to 7 T to reach a maximum value of 30.31 μ_B_ (**1**) at 2 K (Figure S3). The *M* versus *H*/*T* plots (Figure S3, inset) for **1** do not superpose on a single master curve at different temperatures, indicating the presence of low‐lying energy states and/or anisotropy in the system [25, 38, 39, 40, 41, 42, 43]. The magnetization of **2** behaves similarly, reaching a maximum value of 37.7 μ_B_ at 2 K (Figure S4). This would be consistent with a spin state of *S* = 19 to 22. The magnetization of **3** increases almost linearly up to 7 T to reach a maximum value of 8.6 μ_B_ at 2 K (Figure S4).

The temperature‐dependent ac susceptibility measurements on complex **1** reveal the presence of in‐phase (*χ′*) and weak out‐of‐phase (*χ″*) signals below 4 K (Figure S5), indicating slow relaxation of magnetization. Given the weakness of the signal at zero applied dc field, additional χ′ and χ″ versus frequency (Figure S6) measurements were performed under the optimal field of 2 kOe. This leads to the observation of maxima in the χ″ versus frequency plots (Figure S6, right) at lower frequencies (Figures S6–S8).

The temperature dependence of the relaxation times, extracted from these data using a generalized Debye model, was fitted using Equation 1.

(1) $$\tau^{-1}=A\cdot T+\tau_{0}^{-1}\cdot \exp\left(-\frac{U_{\text{eff}}}{k_{B}T}\right)$$

Although the relaxation times can be fitted using only an Orbach process within the experimental error, this leads to a rather slow relaxation rate of *τ*_0_ = 1.4·10^−6^ s. The inclusion of an additional direct process, the consequence of the applied dc field of 2 kOe, improves the fit significantly, resulting in a typical Orbach relaxation rate of *τ*_0_ = 2.4·10^−8^ s and an effective energy barrier of 18.3 K.

### 2.3 MicroSQUID Measurements

MicroSQUID measurements [44] were performed on single crystals of complex **1** at temperatures between 0.04 and 0.8 K and sweep rates between 0.001 and 0.280 T/s. Sweep rate‐independent hysteresis loops at 0.04 K show a coercive field of 0.6 T at a sweep rate of 0.28 T/s (Figures 4 and S9). A correlation between the spin structure and the observed magnetization behavior at 40 mK is provided after the results of the quantum chemical calculations in terms of coupling constants and the relative orientation of the Ni^II^ and Dy^III^ spins.

**FIGURE 4:**
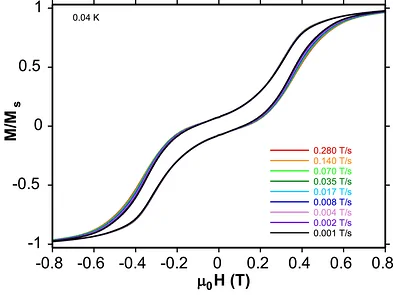
The sweep rate‐dependent hysteresis loops of **1** at 0.04 K.

### 2.4 Quantum Chemical Calculations

In order to assess the magnetic properties of the Ni_8_Ln_4_ compounds, the Y‐analogue (**3**) is considered first to quantify the Ni^II^–Ni^II^ interactions. The possible exchange pathways in **3** as well as in **1** and **2** are shown in Figure S11. For this, the dc magnetic data of **3** were fitted using PHI [45] resulting in a Ni–Ni intradimer interaction (*J*_2_) of +9.1 cm^−1^ and an interdimer interaction (*J*_3_) of −2.36 cm^−1^. Note that here and elsewhere in this paper we use a spin Hamiltonian of the *H* = −2*J*(**S_1_·S_2_**) formalism. This is in good qualitative agreement with the results of DFT calculations, which gave coupling constants of *J*_2_ as +13.2 cm^−1^ and *J*_3_ as −4.4 cm^−1^, respectively. The PHI fitted values are slightly smaller than DFT values; such an overestimation/underestimation is common with DFT‐computed and PHI‐fitted *J* values [46, 47, 48, 49, 50]. We noted earlier that *J*_2_ was expected to be ferromagnetic since the Ni─O─Ni angles at O(6) and O(9) are both less than 90° [37]. The *χT* versus *T* plot from the PHI fitting and that simulated by PHI using the coupling constants obtained from the DFT calculations are each shown as solid lines in Figure 3 bottom. This results in a ground state with *S* = 0, as shown by the DFT calculated spin density plots in Figure S10 (BS2).

We then considered the Gd‐analogue (**2**), which contains paramagnetic and isotropic lanthanide ions. This offers the opportunity to investigate the Ni–Ln coupling without interfering anisotropy, making DFT calculations possible. The different spin configurations in **2** are shown in Figure S11 (top) and Table S4, respectively. For **2**, DFT calculations yielded the Gd^III^–Ni^II^ exchange interaction (*J*_1_) as +0.54 cm^−1^, while the intra (*J*_2_) and interdimer (*J*_3_) Ni^II^–Ni^II^ exchange interactions are similar to those for **1** with +13.4 and −4.41 cm^−1^, respectively. A previous magneto–structural correlation for {GdNi} and {NiGdNi} complexes showed that Gd─O─Ni angles larger than 84° result in ferromagnetic interactions; with Gd─O─Ni angles of 91°, 96°, and 107° associated with Ni–Gd couplings of +0.18, +0.30, and +1.07 cm^−1^, respectively [20]. The Gd─O─Ni angle found here, 103.5°, and the computed *J*_1_ of +0.54 cm^−1^ are consistent with this trend. The DFT‐computed spin–density plots of **2** in Figure S12 indicate a ferromagnetic spin ground state of *S* = 22 for the {NiGd} cyclic structure, highlighting its significant high‐spin character among hetero metallic {NiGd} cyclic assemblies [29, 51, 52, 53, 54, 55, 56, 57].

We are now in a position to assess the magnetic behavior of the Dy‐analogue (**1**). For this, we carried out *ab initio* CASSCF/RASSI‐SO/SINGLE_ANISO calculations using the MOLCAS 8.2 [58] program. The orientation of the anisotropy *g*_zz_ axes in the ground doublets of the Dy^III^ ions and the *D*_zz_ axes in the Ni^II^ ions is depicted in Figure 5.

**FIGURE 5:**
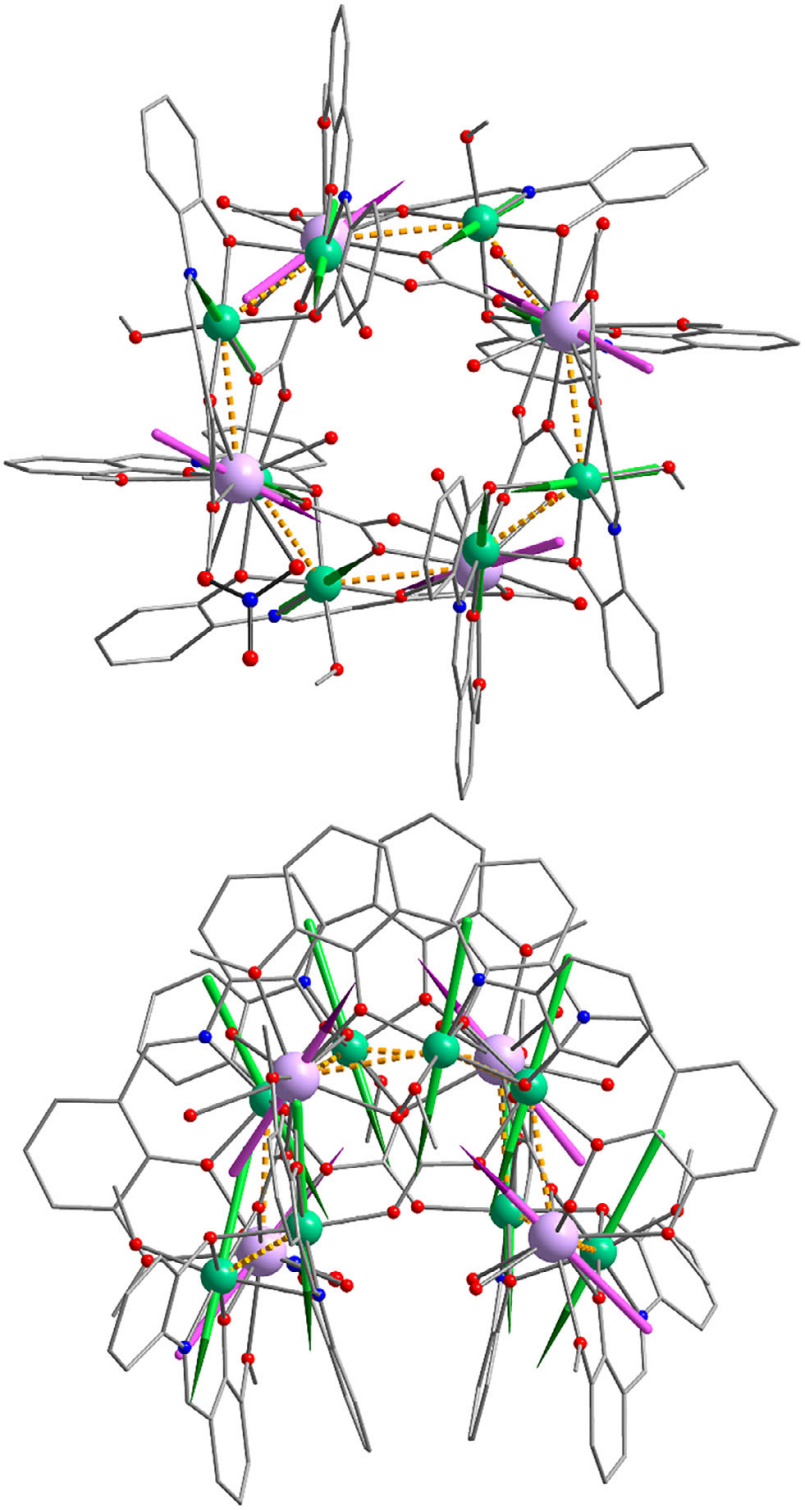
Two views on the anisotropy axes of both Dy^III^ and Ni^II^ ions as computed using SINGLE_ANISO calculations.

This arrangement of axes in the ground state also allows for the rationalization of the micro‐SQUID measurements (Figure 4). At zero field, all Ni^II^ moments are essentially parallel to the four‐fold axis, whereas the Dy^III^ moments have major components pointing along the opposite direction of the four‐fold axis. This results in a net magnetic moment aided by the single‐ion anisotropy of the Dy^III^ ions, leading to open hysteresis. At 0.3 T, an inflection point is observed indicative of a level crossing, which here signals the flipping of the Dy^III^ spins as the magnetic field overcomes the antiferromagnetic Dy–Ni interactions, leading to a steeper increase in magnetization.

To compute the magnetic relaxation of each Dy^III^ and Ni^II^ metal center, we have fragmented complex **1,** considering its extremely large size. The modelled structures are shown in Figure S13. The electronic structure of all four Dy^III^ ions shows that the *g* tensors in the ground KDs have smaller *g*_z_ values than the value of 20 for a pure *m*_J_ = ±15/2 state and large transverse *g*_x_ and *g*_y_ components (Tables 1 and S5). The presence of transverse components indicates that the ground KDs contain highly mixed *m*_J_ states, which is likely to result in efficient QTM.

**TABLE 1:** Low‐lying energies (cm^−1^) and *g*‐tensors of the Dy^III^ fragments that originate from the corresponding ground Kramers Doublets (KDs).

KD	Dy1	Dy2	Dy3	Dy4
**1**	0.0	0.0	0.0	0.0
**2**	45.8	36.0	36.1	36.7
**3**	139.8	127.7	127.8	128.4
**4**	213.3	178.7	178.7	179.7
**5**	283.0	226.6	226.6	228.0
**6**	379.5	299.9	299.9	301.6
**7**	467.4	366.6	366.7	368.5
**8**	570.4	401.2	401.2	403.4
**Ground state KDs**				
*g*_x_	0.4498	0.4427	0.4431	0.4332
*g*_y_	1.9698	1.8436	1.8491	1.7874
*g*_z_	16.9691	18.2526	18.2910	18.3448

To investigate the magnetic relaxation of individual Dy^III^ ions, energies of low‐lying KD states were computed. Tables 1 and S6 list the computed energy gaps between the ground KDs and the excited states for each Dy^III^ ion. The energy gaps to the first excited KDs are 45.8, 36.0, 36.1, and 36.7 cm^−1^ for Dy1‐4, respectively. This reflects the molecular structure with the nitrate localized on Dy1, leading to a larger energy separation between ground and first excited KD but a smaller *g*_z_ component in the ground state compared to the other Dy^III^ ions (Tables 1 and S5 and S6).

Figures 6 and S14 depict a qualitative magnetic relaxation mechanism for Dy1, Dy2, Dy3 and Dy4, respectively. Significant QTM is observed between the ground KDs for the Dy centers, indicating that magnetic relaxation is likely to occur via ground KDs. The presence of QTM in the ground KDs is attributed to the mixing of ±15/2 and ±11/2 states for the Dy1 center and of the ±15/2 and ±3/2 states for Dy2, Dy3, and Dy4 centers. The existence of large QTM in the ground KDs is further validated by computing the crystal‐field parameters using SINGLE_ANISO [58], which results in larger non‐axial terms (*q* ≠ 0 and *k* = 2, 4, 6) compared to the axial terms (*q* = 0 and *k* = 2, 4, 6) (Table S7) [59]. This suggests that negligible slow magnetization relaxation is expected from the individual Dy ions. However, when the Dy ions are coupled with anisotropic Ni ions, the reorientation of magnetization with reasonable barriers is possible. Therefore, the anisotropic nature of Ni ions was verified by computing their axial ZFS values and calculating the exchange interactions between the metal centers.

**FIGURE 6:**
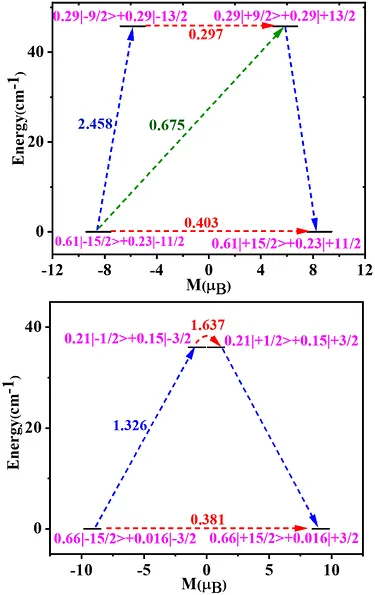
Magnetization blocking barrier for Dy1 (above) and Dy2 (below) in **1**. The barriers for Dy3 and Dy4 are similar to that for Dy2. The black lines indicate the Kramers doublets (KDs). The blue and green arrows show the possible pathway through Orbach and Raman relaxation, respectively. The dotted red lines represent QTM/TA‐QTM. The numbers provided at each arrow are the mean absolute values for the corresponding matrix element of the transition magnetic moment.

The possible QTM arising from each Dy^III^ ion may be suppressed if (i) the Ni^II^ ions exhibit significant anisotropy and (ii) there are substantial magnetic exchange interactions between the Dy^III^ ions and the Ni^II^ ion. To elucidate the impact of the anisotropic behavior of Ni^II^ ions and the exchange interactions on the SMM characteristics of complex **1**, we computed the *D* value of the Ni^II^ ion utilizing the SINGLE_ANISO method and the Dy···Ni exchange interactions employing POLY_ANISO. Our computations predict the *D* values for the Ni^II^ ions in the range of −12.4 to  +9.5 cm^−1^ (Table S8). These values fall within the anticipated range for a Ni^II^ ion in a distorted octahedral shape [20, 60, 61].

Using the POLY_ANISO program, the exchange‐coupled states were developed by considering the total exchange interactions between the Dy–Ni and Ni–Ni ions. The presence of magnetic dipolar interactions (*J*_dipolar_) and exchange interactions (*J*_exch_) have been considered to account for the total magnetic interaction. The latter was included in the Lines model framework [62], and the following equation is used to extract the total exchange interaction:

$$J_{\mathrm{tot}}=J_{\mathrm{dipolar}}+J_{\mathrm{exch}}$$

A satisfactory fit (solid lines) to both susceptibility and magnetization data (Figures 3 and S3) was obtained with the exchange parameters given below. This supports the level of theory used for the calculations [63]. The total exchange interaction (*J*_tot_) of the Dy^III^–Ni^II^ is computed to be −0.61 cm^−1^, whereas the intra (*J*_2_) and interdimer (*J*_3_) Ni^II^–Ni^II^ interactions are calculated to be +13.40(2) and −0.05(2) cm^−1^. It was necessary to include a small intermolecular interaction (*zJ*) with a value of −0.001 cm^−1^. The major contribution to *J*_tot_ of Dy^III^–Ni^II^ thus arises from the dipolar interaction, which is found to be −0.60(2) cm^−1^, whereas the *J*_exch_ is only −0.01(2) cm^−1^. For the Ni^II^–Ni^II^ intradimer interaction (*J*_2_), the major contribution arises from super exchange, which is found to be +18.4(2) cm^−1^, whereas the dipolar interaction is −5.0(2) cm^−1^. In **1**, the two Ni…Ni ions are separated by only 2.902 Å and are bridged by phenoxido groups. In principle, the magnetic dipole–dipole interaction scales as 1/*r* ^3^, such a short metal–metal distance significantly enhances the exchange interaction via through‐space dipolar coupling [30, 31, 32]. It was previously observed that strong dipolar interactions can be responsible for the manifold of low‐lying magnetic states and the observation of any slow relaxation of magnetization in **1** [6, 64]. To verify this further, the *XT* versus T data and magnetization data were simulated with and without including the dipolar coupling. It is clear that the inclusion of the dipolar interactions provides a much better fit to the experimental *XT* and magnetization data (Figures 3 and S3). This confirms that the magnetic properties are strongly dependent on the dipolar exchange interactions [62, 65].

Figure 7 shows the exchange‐coupled states computed using POLY_ANISO, which predicts that **1** is unlikely to show SMM behavior given its large tunneling gap in the ground state, which allows for fast relaxation (Table S9). As we go to the excited states, the tunneling gap started increasing, and it dropped to a lower value at the ninth to tenth excited states, giving an energy barrier of 9.16 cm^−1^ (13.2 K). This prediction is in good agreement with the lack of maxima in the out‐of‐phase component of the ac susceptibility (Figure S5) under a zero‐dc field, but the field‐induced SMM behavior with a *U*_eff_ of 18.3 K for complex **1**. The importance of Ni–Ni and Dy–Ni interactions (mainly due to dipolar coupling) has been further verified by developing the exchange‐coupled state diagram by considering only the Dy–Dy interactions, which yielded a negligible energy barrier (Figure S15). This further confirms the significance of Ni–Ni and Dy–Ni dipolar interactions in the SMM behavior of these complexes.

**FIGURE 7:**
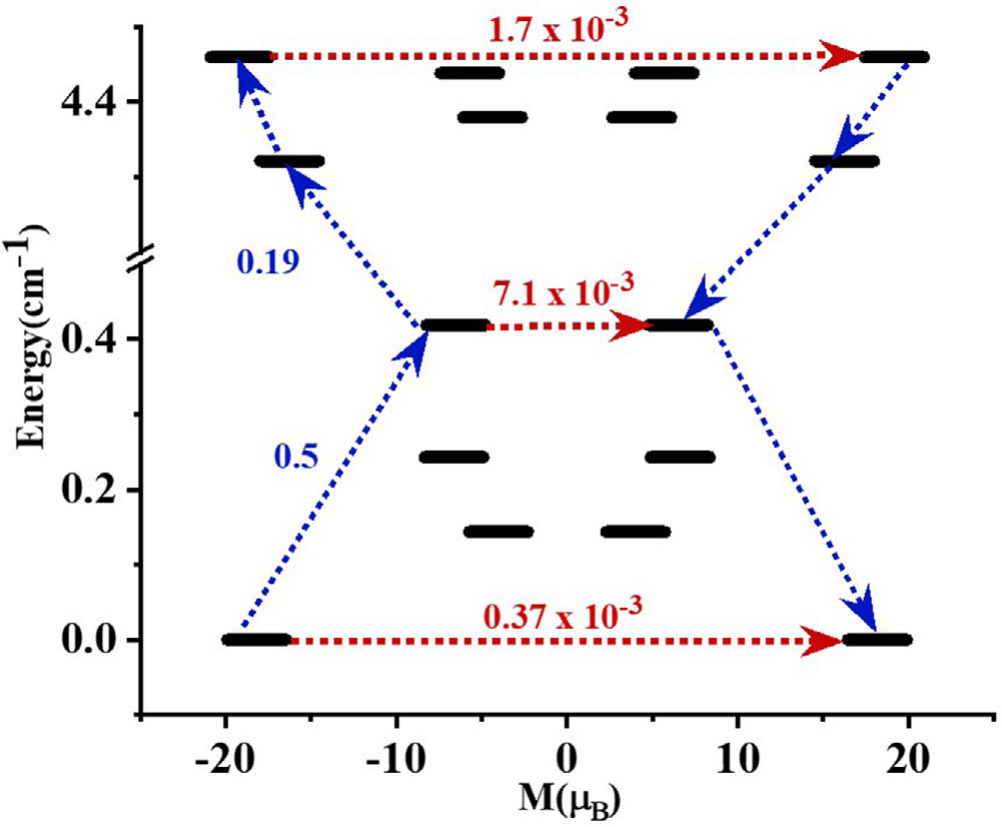
Low‐lying exchange spectrum for **1**. The exchange‐coupled states are placed on the diagram according to their magnetic moments (bold black lines). The blue arrows show the possible pathway via Orbach/Raman relaxation. The solid red lines represent the tunneling gap (Δ_tun_) between the doublet states.

## 3 Conclusion

In conclusion, we have synthesized a carbonate‐containing dodecanuclear Ni_8_Dy_4_ (**1**) coordination cluster and its Gd (**2**) and Y (**3**) analogs. DFT methods were performed on the isostructural complexes **2** and **3** to compute Gd^III^–Ni^II^ and Ni^II^–Ni^II^ interactions. We investigated the spin structure of **1** using quantum chemical methods (*ab initio* CASSCF, SINGLE_ANISO, and POLY_ANISO). We obtained a spin structure with competing Ni–Dy as well as intra‐ and interdimer Ni–Ni interactions. This demonstrates how the transition metal ions steer the spin arrangement of this heterometallic 3d–4f cluster. We used the calculations to rationalize the low temperature magnetic behavior revealed by microSQUID measurements. Furthermore, we could show the importance of dipolar interactions to simulate the experimental magnetic dc data and their importance in terms of the SMM characteristics of **1**.

## Funding

This study was supported by the Deutsche Forschungsgemeinschaft.

## Conflicts of Interest

The authors declare no conflicts of interest.

## Supporting information



The authors have cited additional references within the Supporting Information [66, 67, 68, 69, 70, 71, 72, 73, 74, 75, 76, 77, 78, 79]. Full crystallographic data and details of the structural determination for (**1**) have been deposited with the Cambridge Crystallographic Data Centre as supplementary publication no. CCDC 2555790. Copies of the data can be obtained, free of charge, from https://www.ccdc.cam.ac.uk/structures/.
**Supporting File 1**: chem71499‐sup‐0001‐SupMat.docx

## Data Availability

The data that support the findings of this study are openly available in CCDC at https://www.ccdc.cam.ac.uk/structures/, reference number 2555790.
