# Supplementary material for: A Cyclic {NiII 8DyIII 4} Cluster Where the DyIII Moments Are Organized by the Transition Metal Spins
Source: Chemistry. 2026 Aug 7;32(41):e71499. doi: 10.1002/chem.71499 (PMC13729989; doi:10.1002/chem.71499)
Supplement: Supplementary file 1 [file CHEM-32-e71499-s001.docx]

**Supporting information**

A Cyclic {Ni^II^_8_Dy^III^_4_} Cluster where the Dy^III^ Moments are Organized by the Transition Metal Spins

Imon J. Dutta,^[a],⊥^ Kartik Chandra Mondal,*^[b,c],⊥^ Jonas Braun,^[c,d,e]^ Wolfgang Wernsdorfer,^[e,f]^ Christopher E. Anson,^[c]^ Yanhua Lan,^[c]^ Kuduva R. Vignesh,*^[a]^ and Annie K. Powell*^[c,d,e]^

[a]	I. J. Dutta, Dr. K. R. Vignesh
Department of Chemical Sciences
Indian Institute of Science Education and Research (IISER) Mohali
Sector-81, Knowledge City, S.A.S. Nagar, Mohali-140306, Punjab, India.
E-mail: vigneshkuduvar@iisermohali.ac.in

[b]	Dr. K. C. Mondal
Department of Chemistry
Indian Institute of Technology Madras
Chennai 600036, India.
E-mail: csdkartik@iitm.ac.in

[c]	Dr. K. C. Mondal, Dr. J. Braun, Dr. C. E. Anson, Y. Lan, Prof. Dr. A. K. Powell
Institute of Inorganic Chemistry
Karlsruhe Institute of Technology (KIT)
Kaiserstr. 12, 76131, Karlsruhe, Germany.
E-mail: annie.powell@kit.edu

[d]	Dr. J. Braun, Prof. Dr. A. K. Powell
Institute of Nanotechnology
Karlsruhe Institute of Technology (KIT)
Kaiserstr. 12, 76131, Karlsruhe, Germany.
E-mail: annie.powell@kit.edu

[e]	Dr. J. Braun, Prof. Dr. W. Wernsdorfer, Prof. Dr. A. K. Powell
Institute for Quantum Materials and Technologies
Karlsruhe Institute of Technology (KIT)
Kaiserstr. 12, 76131, Karlsruhe, Germany.
E-mail: annie.powell@kit.edu

[f]	Prof. Dr. Wolfgang Wernsdorfer
Physikalisches Institut
Karlsruhe Institute of Technology (KIT)
Kaiserstr. 12, 76131, Karlsruhe, Germany.

⊥	These Authors contributed equally to this work.

**Synthesis**

The ligand (H_2_L) was synthesized by refluxing the equimolar methanolic solution of *o*-vanillin and 2-aminophenol. The orange-red crystalline ligand was isolated by filtration and utilized for the synthesis of **1**.

**Synthesis of [Ni_8_Dy_4_(CO_3_)_4_(L)_8_(NO_3_)_2_(H_2_O)_8_(MeOH)_4_](NO_3_)_2_·5MeOH·4H_2_O (1):** A mixture of Dy(NO_3_)_3_·6H_2_O (45 mg, 0.1 mmol), Ni(NO_3_)_2_·6H_2_O (58 mg, 0.2 mmol) and H_2_L (48.6 mg, 0.2 mmol) was stirred in 20 mL of MeOH for 5-10 minutes in the presence of Et_3_N (54 mg, 0.54 mmol). The yellow-green solution gradually turned to red-brown upon being left undisturbed under ambient conditions, resulting in red-brown prismatic crystals of **1** after one week with a yield of 70%. Anal. Calc. (%) for C_120_H_138_Dy_4_N_12_Ni_4_O_69_ corresponding to replacement of some MeOH by H_2_O leading to [Ni_8_Dy_4_(CO_3_)_4_(L)_8_(NO_3_)_2_(H_2_O)_8_(MeOH)_4_](NO_3_)_2_·9H_2_O (found): C 36.28 (36.07), N 4.23 (4.15), H 3.50 (3.42). IR (KBr, cm^-1^); 3393(b), 3058(w), 3019(w), 2922(w), 2845(w), 1618(m), 1587(w), 1509(w), 1480(s), 1460(s), 1459(s), 1408(s), 1386(s), 1307(m), 1255(m), 1225(m), 1182(m), 1108(w), 1096(w), 1076(w), 969(w), 823(w), 733(s), 641(w), 576(w), 517(w), 444(w).

**Synthesis of [Ni_8_Gd_4_(CO_3_)_4_(L1)_8_(NO_3_)_2_(H_2_O)_8_(MeOH)_4_](NO_3_)_2_·5MeOH·4H_2_O (2):** The same procedure for **1** was followed for the preparation of **2** using Gd(NO_3_)_3_∙6H_2_O in place of the Dy-salt. Yield: 30%. Anal. Calc. (%) for C_120_H_138_Gd_4_N_12_Ni_4_O_69_ corresponding to replacement of some MeOH by H_2_O leading to [Ni_8_Gd_4_(CO_3_)_4_(L1)_8_(NO_3_)_2_(H_2_O)_8_(MeOH)_4_](NO_3_)_2_·9H_2_O (found): C 36.47 (36.35), N 4.25 (4.18), H 3.52 (3.62). IR (KBr, cm^-1^); 3395(b), 3059(w), 3019(w), 2922(w), 2845(w), 1618(m), 1587(w), 1509(w), 1480(s), 1460(s), 1459(s), 1408(s), 1386(s), 1305(m), 1255(m), 1225(m), 1182(m), 1108(w), 1096(w), 1076(w), 969(w), 823(w), 733(s), 641(w), 576(w), 517(w), 445(w).

**Synthesis of [Ni_8_Y_4_(CO_3_)_4_(L1)_8_(NO_3_)_2_(H_2_O)_8_(MeOH)_4_](NO_3_)_2_·5MeOH·4H_2_O (3):** The same procedure was also followed for the preparation of **3**, using Y(NO_3_)_3_∙6H_2_O. Yield is 30%. Anal. Calc. (%) for C_120_H_138_Y_4_N_12_Ni_4_O_69_ corresponding to replacement of some MeOH by H_2_O leading to [Ni_8_Y_4_(CO_3_)_4_(L1)_8_(NO_3_)_2_(H_2_O)_8_(MeOH)_4_](NO_3_)_2_·9H_2_O (found): C 39.19 (39.15), N 4.57 (4.65), H 3.78 (3.75). IR (KBr, cm^-1^); 3393(b), 3058(w), 3019(w), 2922(w), 2845(w), 1618(m), 1587(w), 1510(w), 1480(s), 1462(s), 1460(s), 1408(s), 1386(s), 1307(m), 1255(m), 1225(m), 1182(m), 1108(w), 1096(w), 1075(w), 969(w), 823(w), 733(s), 641(w), 576(w), 517(w), 445(w).

Whereas the reaction of Dy(NO_3_)_3_·6H_2_O, Ni(NO_3_)_2_·6H_2_O, and H_2_L in 10 mL of DMF in the presence of base (Et_3_N) in the molar ratio of 1:1:2:5 with heating led to the formation of red-brown block-shaped crystals of [(L)_4_Dy_2_Ni_2_(NO_3_)_2_(DMF)_2_] with a defect-dicubane topology after two weeks,^[1]^ using double the amount of Ni^II^ leads to the formation of the title compound Ni_8_Dy_4_ (**1**) with four η^2^: η^1^: η^2^:µ_5_ bridging carbonates (CO_3_^2-^). A proposed mechanism for the formation of **1** is illustrated in Scheme S1.

**Scheme S1**. The proposed mechanism of formation **1**.

**Crystallography**

Data were collected from a crystal of dimensions 0.17 × 0.14 × 0.10 mm^3^, mounted on a Stoe IPDS II diffractometer and cooled to 180(2) K, using Mo-*K*α radiation. The structure was solved by direct methods using SHELXS^[2]^ and refined by full-matrix least-squares refinement using SHELXL-97^[3]^ and SHELXL-2019/3 within Olex2^[4]^ Anisotropic temperature factors were assigned to all non-H atoms, except those corresponding to minor disorder components. Organic H-atoms were placed in calculated positions, whereas the coordinates of H-atoms bonded to ordered O-atoms were refined.

The Ni_8_Ln_4_ clusters all crystallize isomorphously in the tetragonal space group P$\bar{4}$2_1_*c* with Z = 2. The cluster molecule occupies the 2b Wyckoff site with $\bar{4}$ site symmetry. One nitrate counterion is multiply-disordered over the other $\bar{4}$ site (Wyckoff 2a) and so could not be refined satisfactorily; its contribution to the structure factors was calculated using SQUEEZE.^[5]^ Another nitrate counterion occupies a twofold site (Wyckoff 4d, thus two such nitrates per cluster) and apparently undergoes substantial liberational motion, but it could be refined anisotropically using rigid-bond (RIGU) restraints. One water ligand on Dy(1) was ordered, but two other such ligands (together with a lattice water to which they make hydrogen bonds) were disordered against a chelating nitrate, and these could be refined with relative occupancies 75:25, respectively. Thus, although the four Dy centers in the cluster are technically equivalent by the space group crystal symmetry, in any given cluster molecule three of the Dy centers are coordinated by three water ligands, with the fourth having a nitrate and one water ligand. In the crystal, the individual clusters pack with their single nitrate in any of the four possible orientations, and the observed fourfold crystal symmetry is a consequence of this averaging over all molecules.

Crystallographic parameters are summarised in Table S1, where the unit cells for the isomorphous Ni_8_Gd_4_ (**2**) and Ni_8_Y_4_ (**3**) analogues are listed for comparison. Crystals of the latter two compounds were too small to allow for full refinement of their structures. Full crystallographic data and details of the structural determination for (**1**) have been deposited with the Cambridge Crystallographic Data Centre as supplementary publication no. CCDC 2555790. Copies of the data can be obtained, free of charge, from https://www.ccdc.cam.ac.uk/structures/

**Table S1.** Crystallographic Data for the Structure of Ni_8_Dy_4_ (**1**), with the unit cells for the Ni_8_Gd_4_ (**2**) and Ni_8_Y_4_ (**3**) analogues for comparison.

| Compound | **1** | **2** | **3** |
| --- | --- | --- | --- |
| Formula | C_124_H_146_Dy_4_N_12_Ni_8_O_69_ | C_124_H_146_Gd_4_N_12_Ni_8_O_69_ | C_124_H_146_N_12_Ni_8_O_69_Y_4_ |
| Formula weight | 4028.20 | 4007.20 | 3733.84 |
| Crystal System | Tetragonal | Tetragonal | Tetragonal |
| Space Group | P$\bar{4}$2_1_*c* | P$\bar{4}$2_1_*c* | P$\bar{4}$2_1_*c* |
| *a* / Å | 16.8147(9) | 16.8146(14) | 16.8152(15) |
| *c* / Å | 26.0296(19) | 26.0298(18) | 26.030(2) |
| V / Å^3^ | 7359.5(10) | 7359.4(13) | 7360.0(14) |
| Z | 2 | 2 | 2 |
| T / K | 180(2) |  |  |
| *F*(000) | 4028 |  |  |
| *D_c_* / Mg m^-3^ | 1.818 |  |  |
| µ(Mo-*K*α) / mm^-1^ | 3.102 |  |  |
| Data Measured | 42649 |  |  |
| Unique Data | 7825 |  |  |
| *R_int_* | 0.0421 |  |  |
| Data with I ≥ 2σ(I) | 7239 |  |  |
| *wR_2_* (all data) | 0.1073 |  |  |
| *S* (all data) | 1.054 |  |  |
| *R_1_* [I ≥ 2σ(I)] | 0.0421 |  |  |
| Flack χ parameter | -0.037(4) |  |  |
| Parameters/Restraints | 346 / 1 |  |  |
| Biggest diff. peak/hole / eÅ^-3^ | +1.46 / -1.19 |  |  |
| CCDC number | 2555790 |  |  |

**Table S2.** Selected bond lengths (Å) and bond angles (°) within the coordination cluster in **1**.

| Dy1—O4 | 2.320 (5) | Dy1—O1 | 2.456 (4) | Dy1—O5 | 2.547 (6) |
| --- | --- | --- | --- | --- | --- |
| Dy1—O2^i^ | 2.362 (5) | Dy1—O12A | 2.466 (13) | Dy1—O8 | 2.600 (5) |
| Dy1—O11B | 2.367 (17) | Dy1—O12B | 2.477 (13) | Dy1—O11A | 2.516 (17) |
| Dy1—O10 | 2.385 (5) | O2—Dy1^ii^ | 2.361 (5) |  |  |
| Ni1—O1 | 2.059 (4) | Ni1—N1 | 1.989 (6) | Ni1—O14 | 2.089 (5) |
| Ni1—O6 | 2.033 (5) | Ni1—O4 | 2.009 (5) | Ni1—O9^ii^ | 2.142 (5) |
| Ni2—O2^i^ | 2.049 (5) | Ni2—O7 | 1.996 (5) | Ni2—O9 | 2.068 (5) |
| Ni2—O6^i^ | 2.102 (5) | Ni2—N2 | 2.003 (6) | Ni2—O3 | 2.060 (5) |
| Ni1—Ni2 | 2.902 (5) | Ni1—Ni2^i^ | 5.222 (6) | Ni2—Ni2^ii^ | 5.959(6) |
| Ni1—Dy1 | 3.476(6) | Ni2—Dy1 | 3.397(5) | Ni2—Dy1^i^ | 5.622(5) |
| Ni1—O6—Ni2^ii^ | 89.1 (2) | Ni2—O9—Ni1^i^ | 87.12 (17) | Ni2^ii^—O2—Dy1^ii^ | 100.49 (19) |
| Ni1—O4—Dy1 | 106.60 (19) | Ni2—O7—Dy1 | 104.7 (2) | Ni1—O1—Dy1 | 100.31 (16) |
| O10···O3^i^ | 2.797 (7) | O14···O41 | 2.745 (9) | O31···O11A | 2.83 (2) |
| O10···O9^ii^ | 2.729 (8) | O31···O11B | 2.91 (2) |  |  |

Symmetry codes: (i) *y*, −*x*, −*z*+1; (ii) −*y*, *x*, −*z*+1.

The equatorial positions of each Ni^II^ ion are ligated by pocket-I (ONO) (Fig. S1) and one O-atom of CO_3_^2-^ while the axial positions are occupied by one MeOH and one μ-O-atom of iminophenol part of the ligand.

**Figure S1**. (a) Ni^II^ in pocket-I (ONO) and Dy^III^ ion in pocket-II (OO) (top left). (b) Observed bridging modes of L^2-^ (right) and carbonate (CO_3_^2-^, left) in complex **1**. (c) *Syn-syn, syn-anti,* and *anti-anti* bridging modes of carbonate with respect to Ni^II^ ions.

**Figure S2.** Linkages from one Ni_8_Dy_4_ to four others at {*-x, 1-y, z*}, {*1-y, x, 1-z*}, {*x, -1+y, z*} and {*-1+y, -x, 1-z*} *via* hydrogen bonds from MeOH ligands to oxygen atom O(41) of a nitrate counterion.

**DC Magnetic Data**

**Figure S3.** M vs. H plot of complex **1** with and without dipolar interactions (left). Plots of M vs H and M vs H/T (inset) of **1**.

**Figure S4.** Plots of M vs H and M vs H/T (inset) of **2** and **3**.

**AC Magnetic Data**

**Figure S5.** In- and out-of-phase magnetic susceptibility under zero applied magnetic dc field of complex **1**.

**Figure S6.** Field sweep of in- and out-of-phase susceptibility revealing 2 kOe as the optimal field to perform additional ac measurements of complex **1**.

**Figure S7.** In- and out-of-phase susceptibility measured under an applied dc field of 2 kOe. Solid lines are the best fit to a generalized Debye model with α parameters between 0.10 and 0.20.

**Figure S8.** Temperature dependence of the relaxation times fitted using equation 1 (main text). Red line represents fit only taking Orbach relaxation into account; equation 1 can thus be shortened to only use the Arrhenius law term.

**MicroSQUID measurements**

**Figure. S9.** Temperature-dependent hysteresis loops of **1** at indicated temperatures.

**Computational details:**

*Model structures*: The modelled structure to compute the single-ion relaxation of Dy(III) centres possesses a Dy^III^ centre and four Ni^II^ ions replaced by diamagnetic Zn^II^ metal ions. The modelled structure to compute the anisotropic *D* value of Ni^II^ metal ions consists of two Ni centres and a Dy^III^ metal centre, where the neighbouring Ni and Dy ions were replaced by Zn^II^ and Lu^III^ metal ions, respectively. Please see Fig. S13 below. These fragmented models might exclude the cooperative effects, but are not completely neglected throughout the calculations. The cooperative effects have an influence on the local anisotropy of Dy^III^ ions, since the crystal-field interaction is much larger than the exchange interactions between metal centres. Though this was not considered at the single-ion level, the collective magnetic behaviour of the entire cluster is subsequently accounted for by the cooperative effects through the POLY_ANISO calculations using the computed local anisotropy tensors and the exchange/dipolar interactions between the magnetic centres.

*Ab initio calculations Analysis*: Since all Dy^III^ centres are non-equivalent, we have performed the calculations on all four Dy^III^ centres to analyse the direction of the magnetic anisotropy axis using the single-crystal X-ray data. The *ab initio* calculations were performed utilising MOLCAS 8.2^[6]^ on the four Dy^III^ and eight Ni^II^ metal ions. The Douglas-Kroll Hamiltonian is used to account for the relativistic effects.^[7]^ The Complete Active Space Self-Consistent Field (CASSCF) method is used to calculate the spin-free eigenstates.^[8]^ The following basis sets are used for the respective metal ions such as [ANO-RCC... 8s7p5d3f2g1h.] basis set for Dy, [ANO-RCC...7s6p4d3f1g.] for Lu, [ANO-RCC ...6s5p3d2f1g.] for Ni atom, [ANO-RCC...4s3p1d.] for Zn atom, [ANO-RCC...3s2p.] for C atoms, [ANO-RCC...3s2p1d.] for N atoms, [ANO-RCC...3s2p1d.] for O atoms and [ANO-RCC...2s.] for H atoms.^[9]^ MOLCAS-CASSCF calculations are carried out, including nine electrons across seven 4f orbitals of the Dy^III^ metal ion. With this active space, we have computed 21 roots in the Configuration Interaction (CI) procedure. Similarly, the CASSCF calculation included eight electrons across five 3d orbitals of the Ni^II^ ion. With this active space, we computed 10 roots in the triplet and 15 in the singlet Configuration Interaction (CI) procedure to calculate zfs. After calculating all these excited states, we have mixed all roots utilising RASSI-SO^[10]^ and spin-orbit coupling. Furthermore, we have calculated the *g*-tensors with the help of these computed SO states in the SINGLE_ANISO program.^[11]^ Anisotropic *g*-tensors have been computed using eight low-lying Kramers doublets (KDs) of the Dy^III^ ion. The *D*_zz_ axis of Ni centres is also computed using ground doublets. We have calculated the crystal field parameters using the SINGLE_ANISO code as given in MOLCAS 8.2. These computed crystal-field parameters suggest the presence of QTM at the ground states.

*POLY_ANISO Calculations Analysis:* The magnetic susceptibility of complex **1** was simulated using the POLY_ANISO program. Owing to the large nuclearity of the complex, calculations on the full molecule are infeasible. Specifically, the total number of exchange-coupled spin states exceeds 50,000, which is beyond the computational limit of the POLY_ANISO program. To overcome this limitation, the calculations were performed on a reduced magnetic model consisting of one half of the complex, corresponding to a Dy₂Ni₄ core (Figure S16). This model preserves the coordination environments of the magnetic centres and the three exchange pathways, thereby providing a reasonable approximation of the magnetic behaviour of the full complex. Similarly, the other half of the molecule (Dy₂Ni₄ core) was also used for these calculations, which also yielded similar *J* values and a similar profile of an exchange-coupled state mechanism. Since both models gave similar *J* values and mechanisms, the experimentally measured magnetic susceptibility data were reproduced by adding the computed susceptibility data arising from two halves of the magnetic system, and ensuring a meaningful correlation between the computational and experimental susceptibilities.

*ORCA Calculations Analysis:* Cluster anisotropy of the Ni_8_Y_4_ complex was performed using the ORCA suite of programs^[12]^ to calculate the *D*-tensor for the whole molecular system. We employed the DKH-def2-TZVP basis set for the Ni(II) metal ion, the SARC-DKH-TZVPP basis set for the Y(III) ion, the DKH-def2-TZVP(-f) basis set for the O atom, and the DKH-def2-SVP basis set for the rest of the atoms during the zfs calculations.

*DFT Calculations Analysis:* To analyse the exchange interactions between Dy-Ni and Ni-Ni metal centres, we have taken an isotopic system Ni_8_Gd_4,_ which is structurally equivalent to the Ni_8_Dy_4_ system. We have also performed DFT calculations on the Ni_8_Y_4_ to know about the Ni-Ni exchange interaction since the Y^III^ metal ion is diamagnetic.

By utilizing B3LYP functional^[13]^, DFT calculations were performed with the Gaussian 16 suite of programs^[14]^ by replacing Dy^III^ ions with Gd^III^ ions and considering other atom's positions the same as the X-ray-determined structure. We employed the Cundari-Stevens (CS) relativistic effective core potential on Gd^[15]^, TZVP basis set for TM, 6-31G* basis set for C, N, O, H, and LanL2DZ ECP basis set for Y. The magnetic exchange *J* value has been calculated using the Broken Symmetry (BS) method^[16]^ in association with DFT computations. The BS technique has a track record of producing accurate numerical estimates of *J* constants for both simple dinuclear and complicated polynuclear complexes. The exchange interaction (*J*) is calculated using the following Hamiltonian for the {Ni_8_Gd_4_} complex:

$$\hat{\boldsymbol{H}}\boldsymbol{=}-\left[ \boldsymbol{2}\boldsymbol{J}_{\boldsymbol{1}} \left( {\vec{\boldsymbol{S}}}_{\boldsymbol{Gd}\boldsymbol{1}}{\vec{\boldsymbol{S}}}_{\boldsymbol{Ni}\boldsymbol{1}}\boldsymbol{+}{\vec{\boldsymbol{S}}}_{\boldsymbol{Gd}\boldsymbol{2}}{\vec{\boldsymbol{S}}}_{\boldsymbol{Ni}\boldsymbol{3}}\boldsymbol{+}{\vec{\boldsymbol{S}}}_{\boldsymbol{Gd}\boldsymbol{3}}{\vec{\boldsymbol{S}}}_{\boldsymbol{Ni}\boldsymbol{5}}\boldsymbol{+}{\vec{\boldsymbol{S}}}_{\boldsymbol{Gd}\boldsymbol{4}}{\vec{\boldsymbol{S}}}_{\boldsymbol{Ni}\boldsymbol{7}}\boldsymbol{+}{\vec{\boldsymbol{S}}}_{\boldsymbol{Gd}\boldsymbol{1}}{\vec{\boldsymbol{S}}}_{\boldsymbol{Ni}\boldsymbol{8}}\boldsymbol{+}{\vec{\boldsymbol{S}}}_{\boldsymbol{Gd}\boldsymbol{2}}{\vec{\boldsymbol{S}}}_{\boldsymbol{Ni}\boldsymbol{2}}\boldsymbol{+}{\vec{\boldsymbol{S}}}_{\boldsymbol{Gd}\boldsymbol{3}}{\vec{\boldsymbol{S}}}_{\boldsymbol{Ni}\boldsymbol{4}}\boldsymbol{+}{\vec{\boldsymbol{S}}}_{\boldsymbol{Gd}\boldsymbol{4}}{\vec{\boldsymbol{S}}}_{\boldsymbol{Ni}\boldsymbol{6}} \right)\boldsymbol{+2}\boldsymbol{J}_{\boldsymbol{2}} \left( {\vec{\boldsymbol{S}}}_{\boldsymbol{Ni}\boldsymbol{1}}{\vec{\boldsymbol{S}}}_{\boldsymbol{Ni}\boldsymbol{2}}\boldsymbol{+}{\vec{\boldsymbol{S}}}_{\boldsymbol{Ni}\boldsymbol{3}}{\vec{\boldsymbol{S}}}_{\boldsymbol{Ni}\boldsymbol{4}}\boldsymbol{+}{\vec{\boldsymbol{S}}}_{\boldsymbol{Ni}\boldsymbol{5}}{\vec{\boldsymbol{S}}}_{\boldsymbol{Ni}\boldsymbol{6}}\boldsymbol{+}{\vec{\boldsymbol{S}}}_{\boldsymbol{Ni}\boldsymbol{7}}{\vec{\boldsymbol{S}}}_{\boldsymbol{Ni}\boldsymbol{8}} \right)\boldsymbol{+2}\boldsymbol{J}_{\boldsymbol{3}} \left( {\vec{\boldsymbol{S}}}_{\boldsymbol{Ni}\boldsymbol{1}}{\vec{\boldsymbol{S}}}_{\boldsymbol{Ni}\boldsymbol{8}}\boldsymbol{+}{\vec{\boldsymbol{S}}}_{\boldsymbol{Ni}\boldsymbol{2}}{\vec{\boldsymbol{S}}}_{\boldsymbol{Ni}\boldsymbol{3}}\boldsymbol{+}{\vec{\boldsymbol{S}}}_{\boldsymbol{Ni}\boldsymbol{4}}{\vec{\boldsymbol{S}}}_{\boldsymbol{Ni}\boldsymbol{5}}\boldsymbol{+}{\vec{\boldsymbol{S}}}_{\boldsymbol{Ni}\boldsymbol{6}}{\vec{\boldsymbol{S}}}_{\boldsymbol{Ni}\boldsymbol{7}} \right) \right]$$

And for the {Ni_8_Y_4_} complex, the Hamiltonian is:

$$\hat{\boldsymbol{H}}\boldsymbol{=}-\left[ \boldsymbol{2}\boldsymbol{J}_{\boldsymbol{2}} \left( {\vec{\boldsymbol{S}}}_{\boldsymbol{Ni}\boldsymbol{1}}{\vec{\boldsymbol{S}}}_{\boldsymbol{Ni}\boldsymbol{2}}\boldsymbol{+}{\vec{\boldsymbol{S}}}_{\boldsymbol{Ni}\boldsymbol{3}}{\vec{\boldsymbol{S}}}_{\boldsymbol{Ni}\boldsymbol{4}}\boldsymbol{+}{\vec{\boldsymbol{S}}}_{\boldsymbol{Ni}\boldsymbol{5}}{\vec{\boldsymbol{S}}}_{\boldsymbol{Ni}\boldsymbol{6}}\boldsymbol{+}{\vec{\boldsymbol{S}}}_{\boldsymbol{Ni}\boldsymbol{7}}{\vec{\boldsymbol{S}}}_{\boldsymbol{Ni}\boldsymbol{8}} \right)\boldsymbol{+ 2}\boldsymbol{J}_{\boldsymbol{3}} \left( {\vec{\boldsymbol{S}}}_{\boldsymbol{Ni}\boldsymbol{1}}{\vec{\boldsymbol{S}}}_{\boldsymbol{Ni}\boldsymbol{8}}\boldsymbol{+}{\vec{\boldsymbol{S}}}_{\boldsymbol{Ni}\boldsymbol{2}}{\vec{\boldsymbol{S}}}_{\boldsymbol{Ni}\boldsymbol{3}}\boldsymbol{+}{\vec{\boldsymbol{S}}}_{\boldsymbol{Ni}\boldsymbol{4}}{\vec{\boldsymbol{S}}}_{\boldsymbol{Ni}\boldsymbol{5}}\boldsymbol{+}{\vec{\boldsymbol{S}}}_{\boldsymbol{Ni}\boldsymbol{6}}{\vec{\boldsymbol{S}}}_{\boldsymbol{Ni}\boldsymbol{7}} \right) \right]$$

The PHI program^[17]^ was used for fitting the magnetic susceptibilities for the {Ni_8_Y_4_} complex, which uses the *2J* Hamiltonian (above); the same was used as in DFT. A similar Hamiltonian usage at PHI as well as in DFT supports the direct comparison of fitted and simulated *J* values.

**HS**

**BS1**

**BS2**

**Figure S10**. DFT-computed spin-density plots of {Ni_8_Y_4_} complex.

**Table S3.** Different spin configurations employed for extracting *J* values in the {Ni_8_Y_4_} system (**3**).

|  | Y1 | Y2 | Y3 | Y4 | Ni1 | Ni2 | Ni3 | Ni4 | Ni5 | Ni6 | Ni7 | Ni8 | Energy (Hartree) |
| --- | --- | --- | --- | --- | --- | --- | --- | --- | --- | --- | --- | --- | --- |
| HS |  |  |  |  |  |  |  |  |  |  |  |  | **-21217.20583** |
| BS1 |  |  |  |  |  |  |  |  |  |  |  |  | **-21217.20487** |
| BS2 |  |  |  |  |  |  |  |  |  |  |  |  | **-21217.20632** |

**Figure S11.** Magnetic cores with possible exchange interactions **2** (top) and **3** (bottom). Similar exchange pathways of **2** were adopted for **1**.

**Table S4.** Different spin configurations were employed for extracting *J* values in the Ni_8_Gd_4_ system (**2**).

|  | **Gd1** | **Gd2** | **Gd3** | **Gd4** | **Ni1** | **Ni2** | **Ni3** | **Ni4** | **Ni5** | **Ni6** | **Ni7** | **Ni8** | **Energy (Hartree)** |
| --- | --- | --- | --- | --- | --- | --- | --- | --- | --- | --- | --- | --- | --- |
| **HS** |  |  |  |  |  |  |  |  |  |  |  |  | **-21513.57705** |
| **BS1** |  |  |  |  |  |  |  |  |  |  |  |  | **-21513.57688** |
| **BS2** |  |  |  |  |  |  |  |  |  |  |  |  | **-21513.57592** |
| **BS3** |  |  |  |  |  |  |  |  |  |  |  |  | **-21513.57590** |
| **BS4** |  |  |  |  |  |  |  |  |  |  |  |  | **-21513.57737** |
| **BS5** |  |  |  |  |  |  |  |  |  |  |  |  | **-21513.57689** |

**Figure S12.** DFT-computed spin-density plots of {Ni_8_Gd_4_} complex.

(d)

(a)

(b)

(c)

(e)

(f)

(g)

(h)

(i)

(j)

(k)

(l)

**Figure S13.** Model structures for (a) Dy1, (b) Dy2, (c) Dy3, (d) Dy4, (e) Ni1, (f) Ni2, (g) Ni3, (h) Ni4, (i) Ni5, (j) Ni6, (k) Ni7, (l)Ni8.

**Table S5.** *g*-Tensors of the Dy^III^ Fragments That Originate from The Corresponding Ground Kramers Doublets:

| **Kramers doublet** |  | **Dy1** | **Dy2** | **Dy3** | **Dy4** |
| --- | --- | --- | --- | --- | --- |
| **1** | *g*_x_  *g*_y_  *g*_z_ | 0.4498  1.9698  16.9691 | 0.4427  1.8436  18.2526 | 0.4431  1.8491  18.2910 | 0.4332  1.7874  18.3448 |
| **2** | *g*_x_  *g*_y_  *g*_z_ | 0.4325  1.2487  14.0153 | 0.7787  1.0510  17.1083 | 0.7779  1.0523  17.2052 | 0.7228  1.0429  17.2683 |
| **3** | *g*_x_  *g*_y_  *g*_z_ | 1.0277  2.3725  12.8112 | 1.5647  2.5751  14.2774 | 1.5708  2.5843  14.3209 | 1.5736  2.5973  14.3214 |
| **4** | *g*_x_  *g*_y_  *g*_z_ | 8.8344  6.3923  2.9859 | 2.4753  5.3637  10.8210 | 2.4881  5.3542  10.9079 | 2.5294  5.3776  10.8747 |
| **5** | *g*_x_  *g*_y_  *g*_z_ | 3.5793  3.8618  11.0341 | 3.3141  4.3766  11.2190 | 3.3338  4.3967  11.2284 | 3.2796  4.3624  11.2398 |
| **6** | *g*_x_  *g*_y_  *g*_z_ | 0.4711  0.7077  14.8740 | 0.4714  0.8436  15.0059 | 0.4721  0.8490  15.0276 | 0.4745  0.8542  14.9900 |
| **7** | *g*_x_  *g*_y_  *g*_z_ | 0.0964  0.1114  17.4442 | 0.0100  0.4205  17.6043 | 0.0098  0.4228  17.6006 | 0.0062  0.4192  17.5526 |
| **8** | *g*_x_  *g*_y_  *g*_z_ | 0.0207  0.0544  19.2913 | 0.1538  0.4867  18.3487 | 0.1544  0.4879  18.3977 | 0.1520  0.4803  18.3822 |

**Table S6.** RASSI energies of the lowest spin-orbit states (cm^-1^) on each Dy^III^ centre.

| **Dy1** | **Dy2** | **Dy3** | **Dy4** |
| --- | --- | --- | --- |
| 0.0  45.8  139.8  213.3  283.0  379.5  467.4  570.4  3051.3  3079.2  3151.8  3216.0  3295.6  3380.3  3468.7  5654.9  5691.7  5743.9  5827.6  5908.2  6018.9  7850.7  7886.2  7952.0  8054.0  8173.1 9586.9  9621.4  9641.4  9688.7  9719.0  9734.4  9750.5  9792.5  9826.9  9961.3  10981.9  11145.5 11352.1 11840.4  11867.1 11890.7  11922.0 11942.4  13623.1  13659.7 13687.6  13713.8  15029.2  15059.3  15079.2  16036.0  16058.8 16644.2 38805.5  38930.1 39030.5 39120.4 40209.5 40349.6  40658.8 41390.4 41460.6 | 0.0  36.0  127.7  178.7  226.6  299.9  366.6  401.2  3040.3  3075.1  3127.3  3174.9  3224.2  3271.4  3324.0  5636.6 5673.6  5706.7  5770.7  5812.6  5896.7  7825.4  7864.3  7899.2  7975.3  8060.9  9554.1  9583.7  9601.5  9629.4  9656.4  9668.9  9688.3  9723.1  9762.6  9852.6  10977.7 11092.0 11220.2 11794.0  11804.1  11827.2 11857.3 11871.8 13560.5  13599.4  13626.8 13649.1  14966.5  14997.4  15016.4  15977.6  15991.6  16579.0  38816.3  38906.6  38977.4  39020.6  40213.2  40302.2  40494.9  41314.1  41361.7 | 0.0  36.1  127.8  178.7  226.7  299.9  366.7  401.2  3040.4  3075.2  3127.3  3174.9  3224.3  3271.4  3324.0  5636.7  5673.7  5706.8  5770.7  5812.6  5896.7 7825.4  7864.3  7899.2  7975.3  8060.9  9554.1  9583.8  9601.5  9629.4  9656.4  9668.9  9688.4  9723.1  9762.6  9852.6  10977.8 11092.1 11220.2 11794.0  11804.1 11827.2  11857.3 11871.9 13560.5  13599.4  13626.8 13649.2  14966.5 14997.4  15016.4 15977.6 15991.7 16579.0 38816.3  38906.6 38977.5  39020.6  40213.2 40302.2  40494.9 41314.2 41361.7 | 0.0  36.7  128.4  179.7  228.0  301.6  368.5  403.4  3040.7  3075.9  3127.9  3175.8  3225.5  3273.3  3326.4  5637.3  5674.5  5707.5  5771.8  5814.4  5898.7  7826.2  7865.2  7900.2  7976.7  8062.8  9554.9  9584.8  9602.5  9630.8  9657.7  9670.1  9689.7  9724.2  9763.7  9854.5  10978.2 11093.2 11222.3 11795.2  11805.3 11828.4 11858.5 11873.2 13561.8  13600.6  13628.0 13650.4  14967.7  14998.6  15017.7 15978.8 15992.9 16580.3 38816.6  38907.4 38978.8 39022.4 40213.6 40303.3  40497.6 41315.6 41363.5 |

**Table S7.** SINGLE_ANISO computed crystal field parameters for complex **1**. The major

components in the Table are in bold. $B_{k}^{q}$ $B_{k}^{q}$ $B_{k}^{q}$is the crystal field parameter.

| k | q | $B_{k}^{q}$ | $B_{k}^{q}$ | $B_{k}^{q}$ | $B_{k}^{q}$ |
| --- | --- | --- | --- | --- | --- |
|  |  | Dy1 | Dy2 | Dy3 | Dy4 |
| 2 | -2 | 0.37 | -2.31 | **-4.40** | **-5.86** |
|  | -1 | 0.94 | 0.36 | 0.31 | 2.17 |
|  | 0 | **-1.86** | -2.29 | -2.27 | -2.31 |
|  | 1 | -0.16 | **2.99** | **-3.25** | -2.34 |
|  | 2 | **4.01** | **6.16** | **4.89** | **-3.07** |
| 4 | -4 | -0.001 | 0.05 | 0.05 | -0.05 |
|  | -3 | -0.004 | 0.08 | -0.03 | 0.11 |
|  | -2 | -0.006 | -0.01 | -0.04 | -0.07 |
|  | -1 | -0.024 | 0.19 | -0.16 | -0.04 |
|  | 0 | 0.0007 | -0.0065 | -0.0057 | -0.006 |
|  | 1 | -0.01 | 0.11 | -0.14 | -0.22 |
|  | 2 | 0.015 | 0.07 | 0.068 | -0.02 |
|  | 3 | -0.002 | 0.08 | -0.11 | 0.01 |
|  | 4 | -0.0095 | -0.02 | 0.01 | 0.01 |
| 6 | -6 | -0.0002 | -0.0008 | -0.001 | 0.001 |
|  | -5 | 0.00009 | -0.002 | 0.004 | -0.003 |
|  | -4 | -0.0001 | 0.005 | -0.0004 | -0.003 |
|  | -3 | 0.00001 | 0.005 | -0.002 | 0.007 |
|  | -2 | 0.0001 | 0.0006 | 0.001 | 0.002 |
|  | -1 | 0.0001 | 0.0006 | -0.0009 | -0.001 |
|  | 0 | -0.00002 | -0.0002 | -0.0002 | -0.0002 |
|  | 1 | 0.0002 | -0.001 | 0.001 | 0.0004 |
|  | 2 | 0.00003 | -0.002 | -0.002 | 0.001 |
|  | 3 | 0.0001 | 0.004 | -0.007 | 0.0007 |
|  | 4 | 0.00004 | 0.006 | 0.007 | -0.007 |
|  | 5 | -0.00005 | 0.003 | 0.001 | -0.001 |
|  | 6 | -0.0001 | 0.001 | -0.00001 | 0.001 |

**Table S8.** The Computed Zero Field Splitting (*D* in cm^-1^) and *E* values of Ni^II^ metal centers of complex **1**.

| **Metal centers** | ***D*** | ***E*** |
| --- | --- | --- |
| Ni1 | −12.4124 | 3.6254 |
| Ni2 | 9.5018 | −2.8163 |
| Ni3 | −12.4935 | 3.6243 |
| Ni4 | 9.4909 | −2.8892 |
| Ni5 | −12.4941 | 3.6134 |
| Ni6 | 9.5042 | −2.8160 |
| Ni7 | −12.4941 | 3.6137 |
| Ni8 | 9.5084 | −2.8150 |

**Figure S14.** Magnetisation blocking barrier for (a) Dy3, (b) Dy4 centres in **1**. The thick black line indicates the Kramers doublets (KDs). The blue/green arrows show the possible pathway through Orbach/Raman relaxation. The dotted red lines represent the presence of QTM/TA-QTM between the connecting pairs. The numbers provided at each arrow are the mean absolute values for the corresponding matrix element of the transition magnetic moment.

**Figure S15.** Low-lying exchange spectrum for the Dy_4_Zn_8_ system. The exchange-coupled states are placed on the diagram according to their magnetic moments (bold black lines). The blue arrows show the possible pathway via Orbach/Raman relaxation. The solid red lines represent the tunnelling gap (Δ_tun_) between the connecting doublet states.

**Figure S16.** Two Dy_2_Ni_4_ half-systems extracted from **1**, used as models in the POLY_ANISO calculations.

**Table S9.** Lowest exchange doublets (cm^-1^) corresponding to tunnel splitting (Δ_tun_, cm^-1^) and the *g*_z_ value of each doublet (*g*_x_ and *g*_y_ = 0) for complex **1** and energies of the lowest Ising doublets (cm^-1^).

| No. | E (cm^-1^) | Δ_tun_ | *g*_z_ | Energy (cm^-1^) |
| --- | --- | --- | --- | --- |
| 1 | 0.000000000000  0.000371579958 | 3.7 x 10^-4^ | 24.584079438 | 0.00 |
| 2 | 0.415910446328  0.417395922403 | 1 x 10^-3^ | 15.868882655 | 0.42 |
| 3 | 4.363414847472  4.368156862255 | 5 x 10^-3^ | 27.282957173 | 4.37 |
| 4 | 4.597008751461  4.599485628597 | 2 x 10^-3^ | 15.660280168 | 4.60 |
| 5 | 4.884681491032  4.889923563323 | 5 x 10^-3^ | 18.714846122 | 4.89 |
| 6 | 5.026646329273  5.033712936785 | 7 x 10^-3^ | 32.011791244 | 5.03 |
| 7 | 8.681623634459  8.683276466079 | 2 x 10^-3^ | 26.542308562 | 8.68 |
| 8 | 8.936437198885  8.939531853024 | 3 x 10^-3^ | 15.820037178 | 8.93 |
| 9 | 9.024098015623  9.027216902470 | 3 x 10^-3^ | 26.983218305 | 9.02 |
| 10 | 9.071270774440  9.071828464592 | 6 x 10^-4^ | 18.330655420 | 9.07 |
| 11 | 9.161243782961  9.161793706728 | 5 x 10^-4^ | 17.867304608 | 9.16 |
| 12 | 9.393969437055  9.395652762830 | 2 x 10^-3^ | 35.897987714 | 9.39 |
| 13 | 12.845252906337  12.846744975040 | 1 x 10^-3^ | 26.707433726 | 12.85 |
| 14 | 12.889259393953  12.892034508865 | 3 x 10^-3^ | 12.363357945 | 12.89 |
| 15 | 13.338903632657  13.342111171574 | 3 x 10^-3^ | 17.378450577 | 13.34 |
| 16 | 13.377927836842  13.384696216967 | 7 x 10^-3^ | 23.173013852 | 13.38 |
| 17 | 13.423048581117  13.429091576964 | 6 x 10^-3^ | 18.426780207 | 13.43 |
| 18 | 13.583357519006  13.587459656641 | 4 x 10^-3^ | 17.104678514 | 13.58 |
| 19 | 13.707161615103  13.712979036378 | 6 x 10^-3^ | 34.168830741 | 13.71 |
| 20 | 13.727963829273  13.731785963518 | 4 x 10^-3^ | 29.498080824 | 13.72 |

**Computational analysis of SMM behaviour of 3**

Since complex **3** possesses the ground state of S = 8, the complete molecule coupled anisotropy of **3** was calculated using the Orca program^13^, which yielded a cluster anisotropy value equal to 0.003 cm^-1^ and an E/D value of 0.005. It has been found that the major contribution to the *D* value comes from the spin-spin part (0.002 cm^-1^), which also indicates the absence of SMM behaviour in the molecule. The zero-field splitting (*D*) values of Ni metal ions in complex **1** have positive values equal to 9.50 for even no. of metal ions (Ni 2,4,6,8) and possess negative values equal to –12.50 for odd no. of metal ions (Ni 1,3,5,7).

**References:**

[1]	K. C. Mondal, G. E. Kostakis, Y. Lan, W. Wernsdorfer, C. E. Anson, A. K. Powell, *Inorg. Chem.* **2011**, *50*, 11604-11611.

[2]	G. M. Sheldrick, *Acta Crystallogr. A* **2008**, *64*, 112-122.

[3]	G. M. Sheldrick, *Acta Crystallogr. C* **2015**, *71*, 3-8.

[4]	O. V. Dolomanov, L. J. Bourhis, R. J. Gildea, J. A. K. Howard, H. Puschmann, *J. Appl. Crystallogr.* **2009**, *42*, 339-341.

[5]	A. L. Spek, *Acta Crystallogr. C* **2015**, *71*, 9-18.

[6]	F. Aquilante, J. Autschbach, R. K. Carlson, L. F. Chibotaru, M. G. Delcey, L. De Vico, I. Fdez Galvan, N. Ferre, L. M. Frutos, L. Gagliardi, M. Garavelli, A. Giussani, C. E. Hoyer, G. Li Manni, H. Lischka, D. Ma, P. A. Malmqvist, T. Muller, A. Nenov, M. Olivucci, T. B. Pedersen, D. Peng, F. Plasser, B. Pritchard, M. Reiher, I. Rivalta, I. Schapiro, J. Segarra-Marti, M. Stenrup, D. G. Truhlar, L. Ungur, A. Valentini, S. Vancoillie, V. Veryazov, V. P. Vysotskiy, O. Weingart, F. Zapata, R. Lindh, *J. Comput. Chem.* **2016**, *37*, 506-541.

[7]	B. A. Heß, C. M. Marian, U. Wahlgren, O. Gropen, *Chem. Phys. Lett.* **1996**, *251*, 365-371.

[8]	B. O. Roos, P.-A. Malmqvist, *Phys. Chem. Chem. Phys.* **2004**, *6*, 2919-2927.

[9]	B. O. Roos, R. Lindh, P. A. Malmqvist, V. Veryazov, P. O. Widmark, A. C. Borin, *J. Phys. Chem. A* **2008**, *112*, 11431-11435.

[10]	P. A. Malmqvist, B. O. Roos, B. Schimmelpfennig, *Chem. Phys. Lett.* **2002**, *357*, 230-240.

[11]	L. F. Chibotaru, L. Ungur, *J. Chem. Phys.* **2012**, *137*, 064112.

[12]	F. Neese, *WIREs Comput. Mol. Sci.* **2022**, *12*, e1606.

[13]	A. D. Becke, *J. Chem. Phys.* **1993**, *98*, 5648-5652.

[14]	M. J. Frisch, G. W. Trucks, H. B. Schlegel, G. E. Scuseria, M. A. Robb, J. R. Cheeseman, G. Scalmani, V. Barone, G. A. Petersson, H. Nakatsuji, X. Li, M. Caricato, A. V. Marenich, J. Bloino, B. G. Janesko, R. Gomperts, B. Mennucci, H. P. Hratchian, J. V. Ortiz, A. F. Izmaylov, J. L. Sonnenberg, Williams, F. Ding, F. Lipparini, F. Egidi, J. Goings, B. Peng, A. Petrone, T. Henderson, D. Ranasinghe, V. G. Zakrzewski, J. Gao, N. Rega, G. Zheng, W. Liang, M. Hada, M. Ehara, K. Toyota, R. Fukuda, J. Hasegawa, M. Ishida, T. Nakajima, Y. Honda, O. Kitao, H. Nakai, T. Vreven, K. Throssell, J. A. Montgomery Jr., J. E. Peralta, F. Ogliaro, M. J. Bearpark, J. J. Heyd, E. N. Brothers, K. N. Kudin, V. N. Staroverov, T. A. Keith, R. Kobayashi, J. Normand, K. Raghavachari, A. P. Rendell, J. C. Burant, S. S. Iyengar, J. Tomasi, M. Cossi, J. M. Millam, M. Klene, C. Adamo, R. Cammi, J. W. Ochterski, R. L. Martin, K. Morokuma, O. Farkas, J. B. Foresman, D. J. Fox, Wallingford, CT, **2016**.

[15]	T. R. Cundari, W. J. Stevens, *J. Chem. Phys.* **1993**, *98*, 5555-5565.

[16]	L. Noodleman, *J. Chem. Phys.* **1981**, *74*, 5737-5743.

[17]	N. F. Chilton, R. P. Anderson, L. D. Turner, A. Soncini, K. S. Murray, *J. Comput. Chem.* **2013**, *34*, 1164-1175.
